# Association of Treatment With Nirmatrelvir and the Risk of Post–COVID-19 Condition

**DOI:** 10.1001/jamainternmed.2023.0743

**Published:** 2023-03-23

**Authors:** Yan Xie, Taeyoung Choi, Ziyad Al-Aly

**Affiliations:** 1 Clinical Epidemiology Center, Research and Development Service, VA St Louis Health Care System, St Louis, Missouri; 2 Veterans Research and Education Foundation of St Louis, St Louis, Missouri; 3 Department of Medicine, Washington University School of Medicine, St Louis, Missouri; 4 Nephrology Section, Medicine Service, VA St Louis Health Care System, St Louis, Missouri; 5 Institute for Public Health, Washington University in St Louis, St Louis, Missouri

## Abstract

**Importance:**

Post–COVID-19 condition (PCC), also known as long COVID, affects many individuals. Prevention of PCC is an urgent public health priority.

**Objective:**

To examine whether treatment with nirmatrelvir in the acute phase of COVID-19 is associated with reduced risk of PCC.

**Design, Setting, and Participants:**

This cohort study used the health care databases of the US Department of Veterans Affairs (VA) to identify patients who had a SARS-CoV-2 positive test result between January 3, 2022, and December 31, 2022, who were not hospitalized on the day of the positive test result, who had at least 1 risk factor for progression to severe COVID-19 illness, and who had survived the first 30 days after SARS-CoV-2 diagnosis. Those who were treated with oral nirmatrelvir within 5 days after the positive test (n = 35 717) and those who received no COVID-19 antiviral or antibody treatment during the acute phase of SARS-CoV-2 infection (control group, n = 246 076) were identified.

**Exposures:**

Treatment with nirmatrelvir or receipt of no COVID-19 antiviral or antibody treatment based on prescription records.

**Main Outcomes and Measures:**

Inverse probability weighted survival models were used to estimate the association of nirmatrelvir (vs control) with post–acute death, post–acute hospitalization, and a prespecified panel of 13 post–acute COVID-19 sequelae (components of PCC) and reported in relative scale as relative risk (RR) or hazard ratio (HR) and in absolute scale as absolute risk reduction in percentage at 180 days (ARR).

**Results:**

A total of 281 793 patients (mean [SD] age, 61.99 [14.96]; 242 383 [86.01%] male) who had a positive SARS-CoV-2 test result and had at least 1 risk factor for progression to severe COVID-19 illness were studied. Among them, 246 076 received no COVID-19 antiviral or antibody treatment during the acute phase of SARS-CoV-2 infection, and 35 717 received oral nirmatrelvir within 5 days after the positive SARS-CoV-2 test result. Compared with the control group, nirmatrelvir was associated with reduced risk of PCC (RR, 0.74; 95% CI, 0.72-0.77; ARR, 4.51%; 95% CI, 4.01-4.99), including reduced risk of 10 of 13 post–acute sequelae (components of PCC) in the cardiovascular system (dysrhythmia and ischemic heart disease), coagulation and hematologic disorders (pulmonary embolism and deep vein thrombosis), fatigue and malaise, acute kidney disease, muscle pain, neurologic system (neurocognitive impairment and dysautonomia), and shortness of breath. Nirmatrelvir was also associated with reduced risk of post–acute death (HR, 0.53; 95% CI, 0.46-0.61); ARR, 0.65%; 95% CI, 0.54-0.77), and post–acute hospitalization (HR, 0.76; 95% CI, 0.73-0.80; ARR, 1.72%; 95% CI, 1.42-2.01). Nirmatrelvir was associated with reduced risk of PCC in people who were unvaccinated, vaccinated, and boosted, and in people with primary SARS-CoV-2 infection and reinfection.

**Conclusions and Relevance:**

This cohort study found that in people with SARS-CoV-2 infection who had at least 1 risk factor for progression to severe disease, treatment with nirmatrelvir within 5 days of a positive SARS-CoV-2 test result was associated with reduced risk of PCC across the risk spectrum in this cohort and regardless of vaccination status and history of prior infection; the totality of findings suggests that treatment with nirmatrelvir during the acute phase of COVID-19 may reduce the risk of post–acute adverse health outcomes.

## Key Points

**Question:**

Is treatment with nirmatrelvir in the acute phase of SARS-CoV-2 infection associated with a lower risk of post–COVID-19 condition (PCC)?

**Findings:**

In this cohort study of 281 793 people with SARS-CoV-2 infection who had at least 1 risk factor for progression to severe COVID-19 illness, compared with 246 076 who had no treatment, nirmatrelvir use in the acute phase (n = 35 717) was associated with reduced risk of PCC, including reduced risk of 10 of 13 post–acute sequelae in various organ systems, as well as reduced risk of post–acute death and post–acute hospitalization. Nirmatrelvir was associated with reduced risk of PCC in people who were unvaccinated, vaccinated, and boosted, and in people with primary SARS-CoV-2 infection and reinfection.

**Meaning:**

In people with SARS-CoV-2 infection and at least 1 risk factor for progression to severe COVID-19 illness, treatment with nirmatrelvir during the acute phase of COVID-19 was associated with reduced risk of PCC.

## Teaser

This cohort study examines the association of treatment with nirmatrelvir in the acute phase of COVID-19 and risk of post–COVID-19 condition.

## Introduction

*Post–COVID-19 condition* (PCC), also known as *long COVID*, is the disease encompassing the post–acute sequelae of SARS-CoV-2 infection, and it affects millions of people around the world.^1,2,3^ Despite PCC affecting a substantial portion of the patient population, there is no approved medication for the prevention or treatment of PCC. Several hypotheses have been proposed to explain the underlying mechanisms of PCC including persistence of the virus (or its fragments) or intensity of the inflammation during the acute phase of the disease.^4^ The antiviral nirmatrelvir (in combination with ritonavir, marketed under the name Paxlovid) that has been shown to reduce the risk of progression to severe acute COVID-19 has been suggested as a candidate drug that may reduce the risk of developing PCC.^5,6^ In December 2021, oral nirmatrelvir was approved in the US for the treatment of acute SARS-CoV-2 infection (typically within 5 days of symptom onset) in nonhospitalized people at risk of progression to severe COVID-19 illness. Millions of people in the US have since received treatment with nirmatrelvir. Urgent calls have been made to evaluate whether treatment with nirmatrelvir in the acute phase of COVID-19 reduces the risk of PCC—but data have thus far been lacking.^5^ Addressing this question will guide treatment approaches of SARS-CoV-2 infections and will inform the effort to develop and optimize prevention and treatment strategies for PCC.

In this cohort study, we used the health care databases of the US Department of Veterans Affairs (VA) to identify patients who had a SARS-CoV-2 positive test result between January 3, 2022, and December 31, 2022, who were not hospitalized on the day of the positive test, who had at least 1 risk factor for progression to severe COVID-19 illness, and who had survived the first 30 days after SARS-CoV-2 diagnosis. We identified those who were prescribed oral nirmatrelvir within 5 days after the positive test and did not receive other outpatient COVID-19 antiviral or antibody treatment within 30 days after the positive test (nirmatrelvir group, n = 35 717) and those who received no outpatient COVID-19 antiviral or antibody treatment within 30 days after the positive test (control group, n = 246 076). We then used the inverse probability weighting approach to balance the characteristics of the groups and evaluate whether treatment with oral nirmatrelvir vs the control was associated with reduced risk of post–acute outcomes, including PCC (from a set of 13 prespecified post–acute sequelae of SARS-CoV-2 infection), post–acute death, post–acute hospitalization, and each individual post–acute sequela.

## Methods

### Setting

The VA operates the largest integrated health care system in the US; the system comprises 1283 health care facilities (including 171 VA medical centers and 1112 outpatient sites) located across the US. The VA provides comprehensive health care to discharged veterans of the US armed forces including preventative and health maintenance, outpatient care, inpatient hospital care, prescriptions, mental health care, home health care, primary care, specialty care, geriatric and extended care, medical equipment, and prosthetics.

### Data Sources

The cohort study was conducted using the VA health care databases. The VA health care data are updated daily and include individual-level demographic information and data on health care encounters, comorbidities, procedures, and surgeries. Data domains included outpatient encounters, inpatient encounters, inpatient and outpatient medications, laboratory results and non-VA care program integrity tools were used. The VA COVID-19 Shared Data Resource was used to collect information on patients with COVID-19 and vaccination status. The Area Deprivation Index (ADI)—which is a composite measure of income, education, employment, and housing—was used as a summary measure of contextual disadvantage at participants’ residential locations.^7^

### Cohort

A flowchart and a timeline of cohort construction are provided in eFigures 1 and 2 in Supplement 1, respectively. There were 332 256 participants who had a positive SARS-CoV-2 test result between January 3, 2022, and December 31, 2022, when their first date of positive test was set to be T_0_. A total of 37 466 participants prescribed nirmatrelvir within 5 days of T_0_ were selected into the nirmatrelvir group. We then selected 36 641 participants with at least 1 risk factor of progression to severe acute COVID-19 illness, which included being older than 60 years, a body mass index (BMI) of greater than 25 (calculated as weight in kilograms divided by height in meters squared), current smoker, cancer, cardiovascular disease, kidney disease, chronic lung disease, diabetes, immune dysfunction, and hypertension. We also excluded participants with liver disease, end-stage kidney disease, estimated glomerular filtration rate (eGFR; a measure of kidney function) of less than 30 mL/min/1.73m^2^ (to convert to mL/s/m^2^, multiply by 0.0167), and/or prescription fill of a medication that precluded them from receiving nirmatrelvir (n = 35 815). Participants who did not use other outpatient COVID-19 antiviral or antibody treatments within 30 days after T_0_ were selected (n = 35 776). To examine the post–acute events, only participants alive 30 days after T_0_ were included in the nirmatrelvir group (final n = 35 717).

A control group of participants was constructed from the 332 256 participants who had a positive SARS-CoV-2 test result between January 3, 2022, and December 31, 2022, when their first date positive test was set to be T_0_. From those who were not prescribed nirmatrelvir within 5 days of T_0_ (n = 294 790), we selected 278 965 participants with at least 1 risk factor of progression to severe acute COVID-19 illness, and excluded participants with liver disease, end stage kidney disease, eGFR of less than 30 mL/min/1.73m^2^, and/or prescription fill of a medication that precluded them from receiving nirmatrelvir (n = 260 093). Participants who did not use any outpatient COVID-19 antiviral or antibody treatments within 30 days after T_0_ (n = 249 443) were further selected. To examine the post–acute events, only participants alive 30 days after T_0_ were included in the control group (final n = 246 076).

The final cohort consisted of 281 793 participants, of which 35 717 were in the nirmatrelvir group and 246 076 in the control group. The cohort was followed-up until February 2, 2023.

### Exposure and Outcomes

We defined exposure as a filled nirmatrelvir prescription within 5 days of SARS-CoV-2 positive test result. We examined the risk of post–acute death and hospitalization and a composite outcome of death or hospitalization. We also studied individual post–acute sequelae—which were selected based on prior evidence^1,2,8,9,10,11,12,13,14,15,16,17,18,19,20,21,22,23^—including ischemic heart disease, dysrhythmia, deep vein thrombosis, pulmonary embolism, fatigue and malaise, liver disease, acute kidney injury, muscle pain, diabetes, neurocognitive impairment, dysautonomia, and shortness of breath and cough. Individual sequelae were defined based on inpatient and outpatient *International Statistical Classification of Diseases and Related Health Problems, Tenth Revision (ICD-10)* diagnosis codes and laboratory results; death was defined based on vital status data; and hospitalization was defined based on inpatient encounter data. The PCC score was built by assigning weights to each individual sequela following the methods of the Global Burden of Disease Long COVID Collaborators (weights provided at https://github.com/yxie618/Nirmatrelvir_PASC)^24^; we then constructed the PCC score for each participant as the sum of weights of all the incident sequelae in that participant during follow up.^25^ Incident outcomes were assessed within those without history of the related outcome within 3 years before T_0_. All outcomes were ascertained 30 days after T_0_.

### Covariates

We identified baseline characteristics that may be associated with the use of treatment and the occurrence or assessment of outcomes based on literature review and prior knowledge.^1,19,25,26^ All covariates were assessed within 3 years before study enrollment unless otherwise specified. Predefined covariates included age, race (White, Black, and other), ethnicity (Hispanic and non-Hispanic), sex, ADI, BMI, smoking status (current, former, and never), history of SARS-CoV-2 infection, use of steroids, use of long-term care, eGFR, systolic and diastolic blood pressure, cancer, chronic lung disease, dementia, diabetes, hyperlipidemia, and immune dysfunction. We adjusted for medications that would have drug-drug interaction with nirmatrelvir-ritonavir based on 3 levels (require temporary hold of concomitant medication; require adjustment of concomitant medication dosing; and require monitoring for adverse effects). We also considered health care utilization parameters including number of outpatient and inpatient encounters, number of laboratory encounters and number of outpatient medications received within 1 year before study enrollment and influenza vaccination status. Continuous variables were transformed into restricted cubic spline functions to account for potential nonlinear associations.

### Statistical Analysis

Baseline characteristics were reported as mean and standard deviation or frequency and percentage. Covariate balance between groups was evaluated by the absolute standardized differences where an absolute standardized difference of less than 0.1 was considered evidence of good balance.

To examine the risk of incident outcomes, for each outcome besides death or hospitalization, we conducted analysis on a subcohort of participants without the history of the outcome within 3 years before T_0_. An inverse probability weighting method was used to balance the differences in baseline characteristics between the nirmatrelvir and control groups. Logistic regression was built to estimate the probability of receiving nirmatrelvir given covariates. The probability was then used as the propensity score. We then constructed the inverse probability weights as a value of 1 for those in the nirmatrelvir group and as propensity score divided by (1−propensity score) for those in the control group to estimate the association within population with same baseline characteristics as the nirmatrelvir group. Weights larger than 10 would be truncated at 10 to reduce the influence of extreme weights (in the present study, no weights were larger than 10, and none were truncated). The inverse probability weights were then applied to a Cox survival model in order to estimate the association of nirmatrelvir with individual outcomes. Hazard ratio (HR) and survival probability for both groups at 180 days were estimated. Absolute risk reduction (ARR) at 180 days was computed as the difference of survival probability in the nirmatrelvir group compared with the control group. To estimate the association of nirmatrelvir with PCC score, the inverse probability weighted zero inflated Poisson regression was used, and the relative risk (RR) and ARR in percentage at 180 days were estimated.

The association of nirmatrelvir with the risk of PCC was further examined within prespecified subgroups by age (≤60 years, >60 years to ≤70 years, and >70 years), race (White and Black), sex, smoking status (current smoker, former smoker and never smoker), cancer, cardiovascular disease, chronic kidney disease, chronic lung disease, diabetes, immune dysfunction, and hypertension. We also examined the association within populations with different vaccination status (unvaccinated, 1-2 doses of vaccine, and boosted) and infection status (with primary SARS-CoV-2 infection and reinfection). To examine the association within populations with different baseline risks, we also defined subgroups based on the number of baseline risk factors (1-2, 3-4, or ≥5) of progression to severe acute COVID-19 illness, where risk factors included age of older than 60 years, BMI greater than 25, current smoker, cancer, cardiovascular disease, kidney disease, chronic lung disease, diabetes, immune dysfunction, and hypertension.

We challenged the robustness of findings in multiple sensitivity analyses, including (1) application of the overlap weighting method to balance baseline characteristics in the treatment and control groups (whereas, in the primary approach, we used the inverse probability weighing approach to balance the groups); (2) application of the doubly robust approach to additionally adjust for covariates in the inverse probability weighted survival models (whereas, in the primary approach, we used inverse probability weighted survival models); (3) application of the high-dimensional variable selection algorithm to additionally identify 100 covariates from data domains including diagnoses, medications, and laboratory test results that were used along with predefined variables to construct the weights (whereas, in the primary approach, we used only predefined covariates); (4) application of inverse probability of censoring weight to account for those who died during the acute phase of infection (within 30 days of infection) (whereas, in the primary approach, we removed those who died during acute phase from the analyses); (5) defined outcomes based on events that occurred 90 days after infection (whereas, in the primary approach, outcomes were defined based on events that occurred 30 days after infection); (6) defined incident outcome as occurrence of the outcome in those without history of the related outcome within 5 years before infection (whereas, in the primary approach, the washout period was 3 years before infection); (7) additionally adjusted for hospitalization, intensive care unit admission and ventilator use during the acute phase of infection (whereas, in the primary approach, we did not adjust for information after exposure); (8) defined outcomes based on PCC *ICD-10* code U09.9 (whereas, in the primary definition, this was based on a set of 13 predefined post–acute sequelae of COVID-19); and (9) defined outcomes based on the first occurrence of any individual sequela (whereas, in the primary definition, this accounted for both the number of sequelae and the influence of each sequela on health).

Analyses were performed with SAS Enterprise Guide, version 8.2 (SAS Institute). Data visualizations were performed in R 4.0.4 (R Project for Statistical Computing). The robust sandwich variance estimator was used to estimate variance in weighted analyses. Risk on relative scale with a 95% CI that does not cross 1 and risk on absolute scale with a 95% CI that does not cross 0 was considered statistically significant. The study was approved by the VA St Louis Health Care System institutional review board, which granted a waiver of informed consent because of the retrospective nature of the study. The study followed the Strengthening the Reporting of Observational Studies in Epidemiology (STROBE) reporting guideline.

## Results

The cohort included 281 793 participants; 35 717 were in the nirmatrelvir group, and 246 076 were in the control group that received no COVID-19 antiviral or antibody treatment within the first 30 days after infection. The demographic and health characteristics before weighting are provided in eTable 1 in Supplement 1; characteristics after weighting are provided in Table 1. Absolute standardized mean differences between the the nirmatrelvir group and the control group after application of inverse probability weighting were all below 0.1—suggesting good balance (Table 1). The unadjusted event rates are presented in eTable 2 in Supplement 1.

**Table 1.** Demographic and Health Characteristics of the Overall Cohort, the Nirmatrelvir Group, and the Control Group After Weighting

Characteristic	No. (%)			SMD between nirmatrelvir and control group^a^
	Overall cohort (n = 281 793)	Control group (n = 246 076)	Nirmatrelvir group (n = 35 717)	
Age, mean (SD), y	65.70 (13.39)	65.76 (13.39)	65.64 (13.38)	0.01
Race				
Black	57 768 (20.50)	50 465 (20.51)	7316 (20.48)	0.001
White	208 696 (74.06)	182 367 (74.11)	26 431 (74.00)	0.002
Other^b^	15 358 (5.45)	13 246 (5.38)	1970 (5.52)	0.006
Ethnicity				
Hispanic	22 121 (7.85)	19 169 (7.79)	2823 (7.90)	0.004
Non-Hispanic	259 672 (92.15)	226 907 (92.21)	32 894 (92.10)	0.004
Sex				
Female	34 294 (12.17)	29 741 (12.09)	4378 (12.26)	0.005
Male	247 499 (87.83)	216 335 (87.91)	31 339 (87.74)	0.005
Smoking status				
Never	121 622 (43.16)	105 825 (43.01)	15 470 (43.31)	0.006
Former	116 719 (41.42)	102 289 (41.57)	14 744 (41.28)	0.006
Current	43 452 (15.42)	37 965 (15.43)	5503 (15.41)	0.001
Area Deprivation Index, mean (SD)^c^	51.07 (19.70)	51.07 (19.74)	51.07 (19.67)	<0.001
Long-term care	1873 (0.64)	1584 (0.64)	289 (0.64)	<0.001
Vaccination				
Without prior vaccination	47 144 (16.73)	41 129 (16.71)	5982 (16.75)	0.001
With 1 shot of vaccination	10 173 (3.61)	8861 (3.60)	1296 (3.63)	0.002
With 2 shots of vaccination	60 191 (21.36)	52 372 (21.28)	7655 (21.43)	0.004
With booster	164 285 (58.30)	143 711 (58.40)	20 784 (58.19)	0.004
BMI, mean (SD)	30.86 (6.37)	30.86 (6.44)	30.86 (6.31)	<0.001
eGFR, mean (SD), ml/min/1.73m^2^^d^	78.56 (18.37)	78.53 (18.41)	78.59 (18.34)	<0.001
Blood pressure, mean (SD), mm Hg				
Systolic	134.14 (11.34)	134.16 (11.34)	134.13 (11.34)	<0.001
Diastolic	78.29 (6.97)	78.26 (6.98)	78.33 (6.96)	0.01
History of SARS-CoV-2 infection	47 933 (17.01)	42 069 (17.10)	6046 (16.93)	0.004
Use of steroid	9271 (3.29)	8167 (3.32)	1162 (3.25)	0.004
Medications that would have drug-drug interaction with nirmatrelvir-ritonavir				
On concomitant medication that requires temporary hold	146 138 (51.86)	128 353 (52.16)	18 411 (51.55)	0.01
On concomitant medication that requires dosing adjustment	109 617 (38.90)	96 523 (39.23)	13 780 (38.58)	0.01
On concomitant medication that requires monitoring for adverse events	107 307 (38.08)	946,38 (38.46)	13 467 (37.71)	0.02
Cancer	50 864 (18.05)	44 569 (18.11)	6425 (17.99)	0.003
Chronic lung disease	63 403 (22.50)	55 468 (22.54)	8019 (22.45)	0.002
Dementia	20 120 (7.14)	17 358 (7.05)	2582 (7.23)	0.007
Diabetes type 2	100 318 (35.60)	87 820 (35.69)	12 684 (35.51)	0.004
Cardiovascular disease	83 157 (29.51)	734,09 (29.83)	10 426 (29.19)	0.01
Hyperlipidemia	111 889 (39.66)	97 741 (39.72)	14 148 (39.61)	0.002
Immune dysfunction	15 978 (5.67)	14036 (5.70)	2010 (5.63)	0.003
No. of hospital admissions, mean (SD)^e^	0.16 (0.61)	0.17 (0.61)	0.16 (0.61)	0.01
No. of outpatient visits, mean (SD)^e^	3.20 (1.39)	3.22 (1.39)	3.19 (1.39)	0.02
No. of blood panel tests, mean (SD)^e^	7.69 (8.04)	7.76 (8.10)	7.63 (7.98)	0.02
No. of medications, mean (SD)^e^	10.31 (7.27)	10.38 (7.31)	10.23 (7.23)	0.02
No. of hospital admissions from Medicare	0.03 (0.21)	0.03 (0.21)	0.03 (0.22)	<0.001
No. of outpatient visits from Medicare	0.14 (0.58)	0.14 (0.58)	0.14 (0.58)	<0.001
Influenza vaccine	195 142 (69.25)	170 796 (69.41)	24 676 (69.09)	0.007
Calendar wk of study enrollment, mean (SD)	31.98 (11.75)	32.07 (11.65)	31.89 (11.85)	0.02

Abbreviations: BMI, body mass index (calculated as weight in kilograms divided by height in meters squared); eGFR, estimated glomerular filtration rate; SMD, absolute standardized mean difference.

^a^ Absolute standardized mean differences below 0.1 were considered good balance.

^b^ Other indicates American Indian, Alaskan Native, Asian, Native Hawaiian, and other Pacific Islander.

^c^ Area Deprivation Index is a measure of socioeconomic disadvantage, with a range from low to high disadvantage of 0 to 100.

^d^ To convert to mL/s/m^2^, multiply by 0.0167.

^e^ Data collected within 1 year before study enrollment.

In this study, measurements of risk are provided on both relative and absolute scale: (1) RR or HR of nirmatrelvir in comparison to the control group and (2) ARR in percentage at 180 days; the latter represents the event rate reduction in the nirmatrelvir group compared with the control group at 180 days.

### Risk of PCC

**Figure 1.**
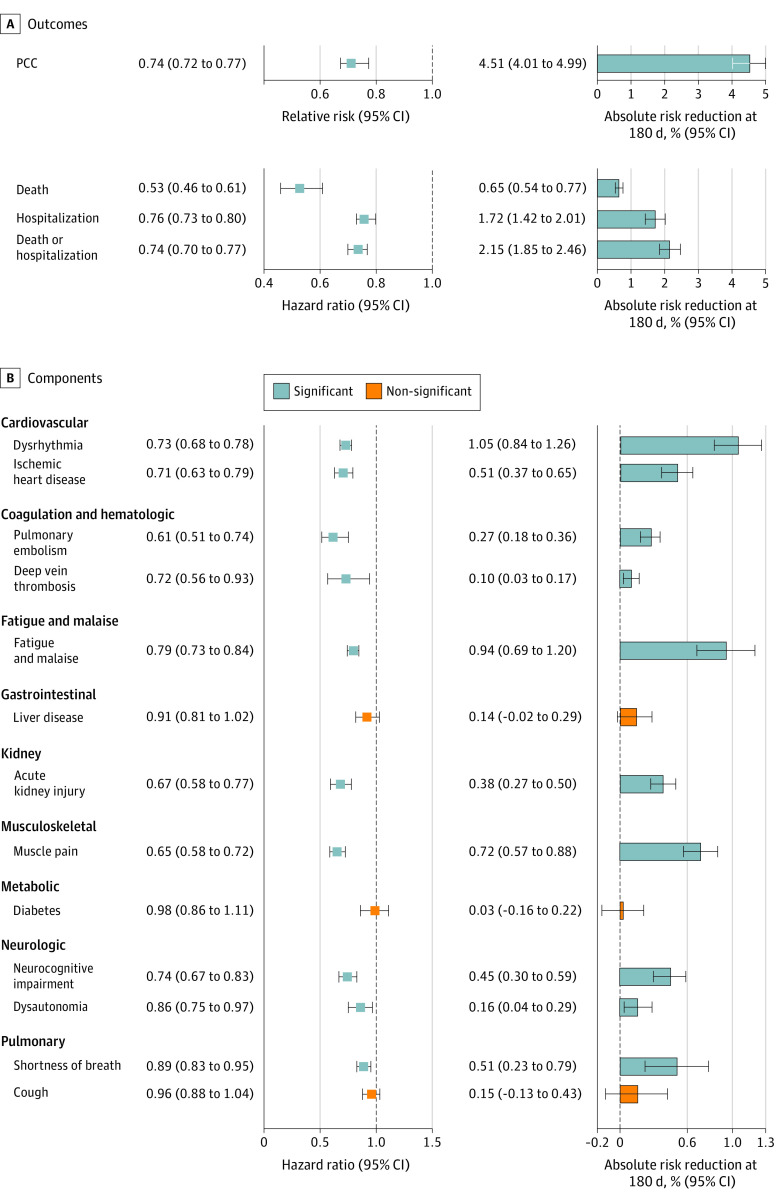
Relative and Absolute Risk Reduction of Nirmatrelvir Compared With the No-Treatment Control Group A, Post–COVID-19 condition (PCC), death, hospitalization, and composite outcome of death or hospitalization. B, Individual post–acute sequelae (components of PCC). Outcomes were ascertained 30 days after the SARS-CoV-2 positive test result until the end of follow-up. The nirmatrelvir group consisted of 35 717 patients, and the control group consisted of 246 076 patients. Adjusted hazard ratios and 95% CIs are presented. Absolute risk reduction of nirmatrelvir compared with the control group per 100 persons at 180 days and associated 95% CIs were estimated based on the difference of survival probability in the nirmatrelvir group compared with the control group. Statistically significant results are presented in light blue, and results that lacked statistical significance are presented in orange.

**Figure 2.**
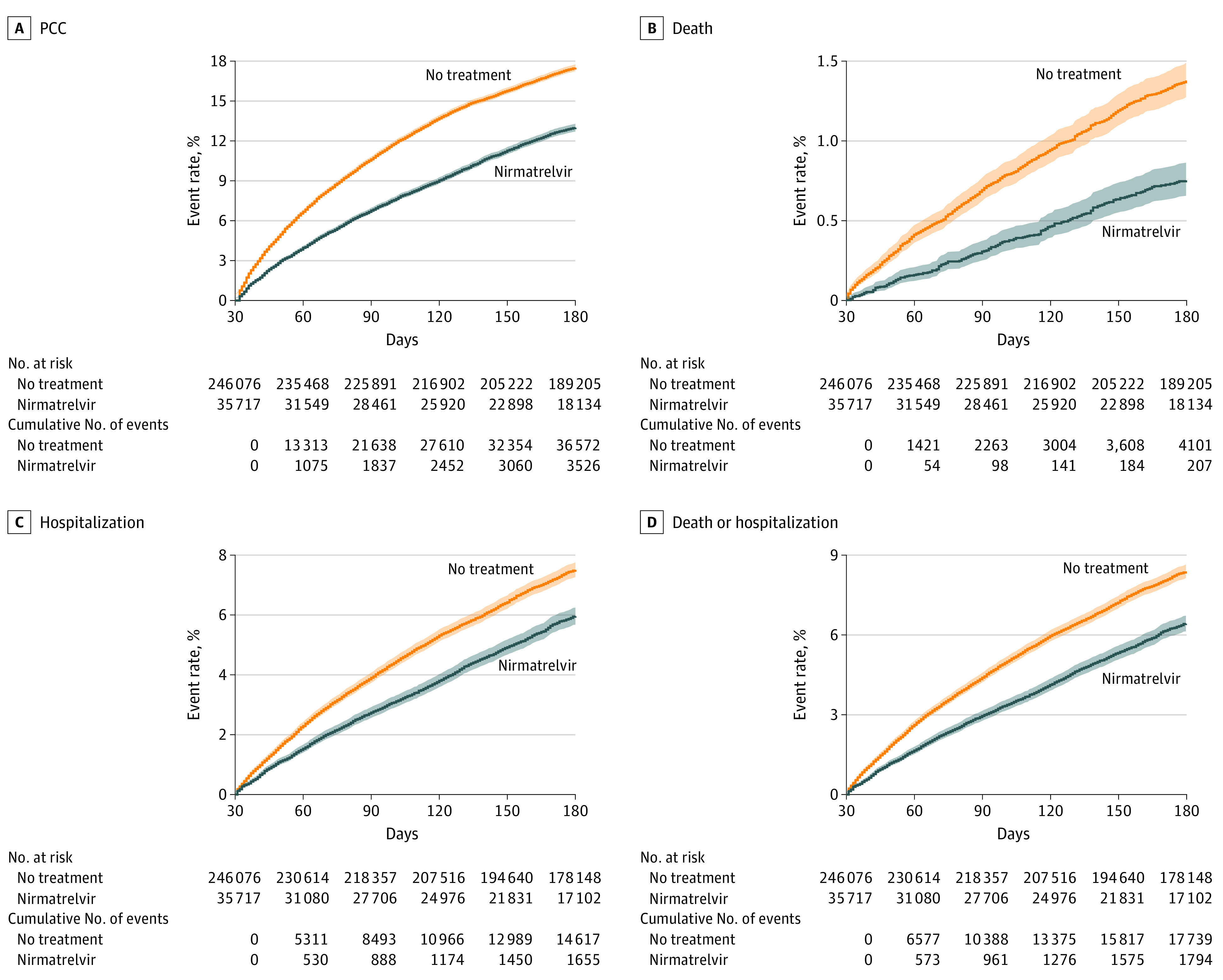
Event Rates of Post–Acute Outcomes in Nirmatrelvir and No-Treatment Control Group A, Post–COVID-19 condition (PCC). B, Death. C, Hospitalization. D, Composite outcome of death or hospitalization. Outcomes were ascertained 30 days after the SARS-CoV-2 positive test until the end of follow-up. Event rates in percentage presented for the nirmatrelvir group (blue, n = 35 717) and the control group (orange, n = 246 076). Shaded areas are 95% CIs.

Compared with the control group, nirmatrelvir was associated with reduced risk of PCC (RR, 0.74; 95% CI, 0.72-0.77); the event rate was 12.99% (95% CI, 12.52-13.49) and 17.51% (95% CI, 17.08-17.94) at 180 days in the nirmatrelvir and the control groups, respectively. This corresponded to an ARR of 4.51% (95% CI, 4.01-4.99) at 180 days (Figures 1A and 2A; eTable 3 in Supplement 1).

### Risk of Post–Acute Death and Post–Acute Hospitalization

Compared with the control group, nirmatrelvir was associated with reduced risk of post–acute death (HR, 0.53; 95% CI, 0.46-0.61; ARR, 0.65%; 95% CI, 0.54-0.77), post–acute hospitalization (HR, 0.76; 95% CI, 0.73-0.80; ARR, 1.72%; 95% CI, 1.42-2.01), and the composite outcome of post–acute death or hospitalization (HR, 0.74; 95% CI, 0.70-0.77; ARR, 2.15%; 95% CI, 1.85-2.46) (Figures 1A and 2B, 2C, and 2D; eTable 3 in Supplement 1).

### Risk of Individual Post–Acute Sequelae

Compared with the control group, nirmatrelvir was associated with reduced risk of 10 of the 13 prespecified post–acute sequelae evaluated in this analysis. Nirmatrelvir was associated with reduced risk of sequelae in the cardiovascular system (dysrhythmia and ischemic heart disease), coagulation and hematologic disorders (pulmonary embolism and deep vein thrombosis), fatigue and malaise, liver disease, acute kidney disease, muscle pain, neurologic system (neurocognitive impairment and dysautonomia), and shortness of breath (Figure 1B, Table 2). There was lack of a statistically significant association between nirmatrelvir and other post–acute sequelae, including new-onset diabetes, liver disease, and cough (Figure 1B, Table 2).

**Table 2.** Hazard Ratio and Absolute Risk Reduction of Nirmatrelvir on Individual Post–Acute Sequelae (Components of Post–COVID-19 Condition) Compared With the Control Group

Organ system	Outcome	Hazard ratio (95% CI)	Event rate, % at 180 d (95% CI)		
			Nirmatrelvir group^a^	Control group^b^	Absolute risk reduction
Cardiovascular	Dysrhythmia	0.73 (0.68 to 0.78)	2.86 (2.67 to 3.06)	3.91 (3.82 to 4.00)	1.05 (0.84 to 1.26)
	Ischemic heart disease	0.71 (0.63 to 0.79)	1.23 (1.10 to 1.36)	1.74 (1.68 to 1.80)	0.51 (0.37 to 0.65)
Coagulation and hematologic	Pulmonary embolism	0.61 (0.51 to 0.74)	0.43 (0.35 to 0.51)	0.70 (0.66 to 0.74)	0.27 (0.18 to 0.36)
	Deep vein thrombosis	0.72 (0.56 to 0.93)	0.26 (0.20 to 0.32)	0.36 (0.33 to 0.39)	0.10 (0.03 to 0.17)
Fatigue and malaise	Fatigue and malaise	0.79 (0.73 to 0.84)	3.53 (3.29 to 3.77)	4.47 (4.37 to 4.58)	0.94 (0.69 to 1.20)
Gastrointestinal	Liver disease	0.91 (0.81 to 1.02)	1.31 (1.17 to 1.45)	1.45 (1.39 to 1.51)	0.14 (−0.02 to 0.29)
Kidney	Acute kidney injury	0.67 (0.58 to 0.77)	0.79 (0.68 to 0.89)	1.17 (1.12 to 1.22)	0.38 (0.27 to 0.5)
Musculoskeletal	Muscle pain	0.65 (0.58 to 0.72)	1.35 (1.22 to 1.49)	2.08 (2.01 to 2.14)	0.72 (0.57 to 0.88)
Metabolic	Diabetes	0.98 (0.86 to 1.11)	1.49 (1.31 to 1.67)	1.52 (1.45 to 1.59)	0.03 (−0.16 to 0.22)
Neurological	Neurocognitive impairment	0.74 (0.67 to 0.83)	1.29 (1.16 to 1.42)	1.74 (1.68 to 1.80)	0.45 (0.30 to 0.59)
	Dysautonomia	0.86 (0.75 to 0.97)	0.97 (0.86 to 1.09)	1.14 (1.09 to 1.19)	0.16 (0.04 to 0.29)
Pulmonary	Shortness of breath	0.89 (0.83 to 0.95)	4.18 (3.91 to 4.44)	4.69 (4.58 to 4.79)	0.51 (0.23 to 0.79)
	Cough	0.96 (0.88 to 1.04)	3.47 (3.21 to 3.73)	3.62 (3.52 to 3.72)	0.15 (−0.13 to 0.43)

^a^ The nirmatrelvir group included those who received a prescription of nirmatrelvir within 5 days after testing positive for SARS-CoV-2 and did not use any other outpatient antiviral or antibody during the first 30 days after a positive test. Outcomes were ascertained 30 days after the SARS-CoV-2 positive test until the end of follow-up.

^b^ The control group included those who did not use any outpatient antiviral or antibody during the first 30 days after testing positive for SARS-CoV-2 and served as the reference group in the analyses. Outcomes were ascertained 30 days after the SARS-CoV-2 positive test until the end of follow-up.

### Risk of PCC in Subgroups

**Figure 3.**
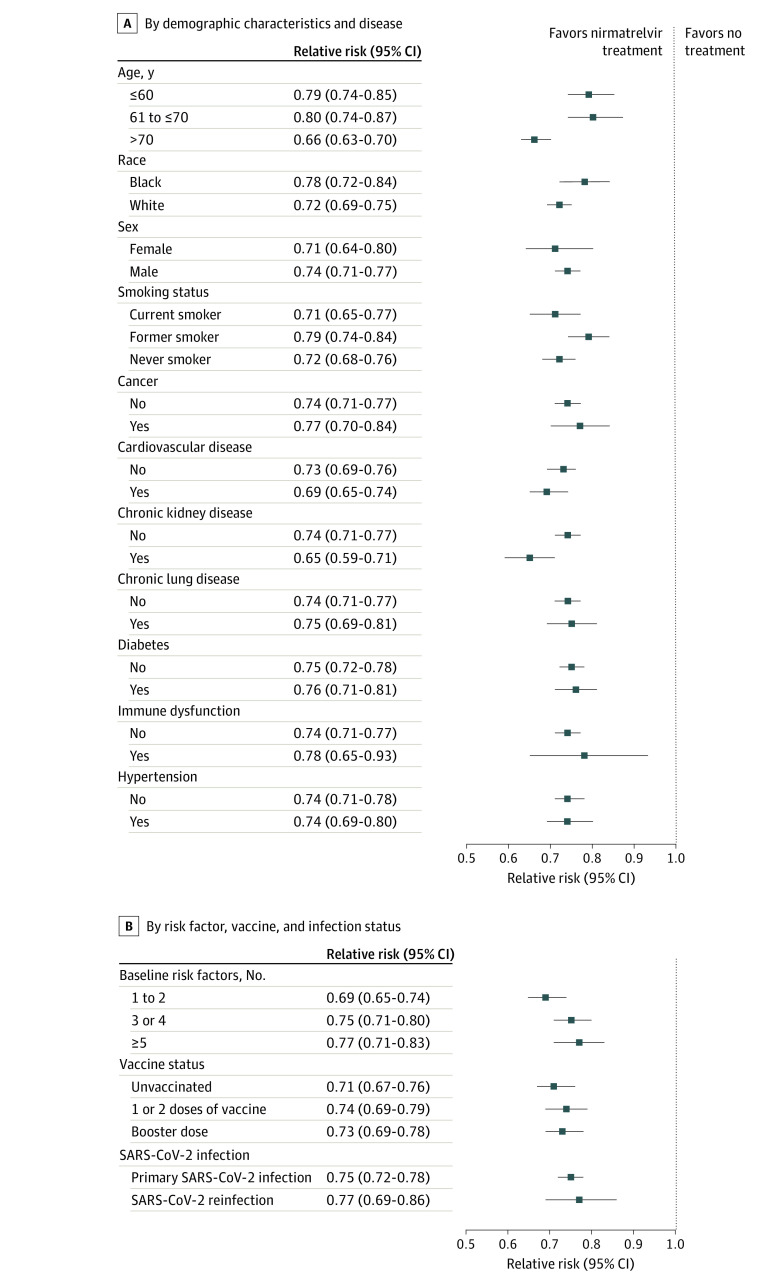
Relative Risk of Post–COVID-19 Condition in the Nirmatrelvir vs No-Treatment Groups A, By demographic and disease subgroups included age (≤60 years, >60 years to ≤70 years, and >70 years), race (White and Black), sex, smoking status (current smoker, former smoker, and never smoker), cancer, cardiovascular disease, chronic kidney disease, chronic lung disease, diabetes, immune dysfunction, and hypertension. B, By number of baseline risk factors (1 to 2, 3 to 4, ≥5), vaccination status (unvaccinated, 1 to 2 doses of vaccine, and boosted), and SARS-CoV-2 infection status (with primary SARS-CoV-2 infection and reinfection). Baseline risk factors of progression to severe acute COVID-19 illness included age of older than 60 years, body mass index of more than 25 (calculated as weight in kilograms divided by height in meters squared), current smoker, cancer, cardiovascular disease, kidney disease, chronic lung disease, diabetes, immune dysfunction, and hypertension. Outcomes were ascertained 30 days after the SARS-CoV-2 positive test until the end of follow-up.

Compared with the control group, people treated with nirmatrelvir exhibited reduced risk of PCC in subgroups based on age, race, sex, smoking, cancer, cardiovascular disease, chronic kidney disease, chronic lung disease, diabetes, immune dysfunction and hypertension (Figure 3A; eTable 4 in Supplement 1).

Because nirmatrelvir is prescribed to people with at least 1 baseline risk factor for progression to severe acute COVID-19 illness, and to better understand the association between nirmatrelvir and the risk of PCC in people with different baseline risk strata, the association between nirmatrelvir and the risk of PCC was tested according to the number of baseline risk factors for progression to severe acute COVID-19 illness. Nirmatrelvir was associated with reduced risk of PCC in people with 1 to 2, 3 to 4, and 5 or more baseline risk factors (Figure 3B; eTable 4 in Supplement 1).

Examination of the association between nirmatrelvir and risk of PCC by vaccine status suggested that nirmatrelvir was associated with reduced risk of PCC in people who were unvaccinated, vaccinated, and those who received a booster vaccine (Figure 3B; eTable 4 in Supplement 1). Nirmatrelvir was associated with reduced risk of PCC in people with primary SARS-CoV-2 infection and in people with reinfection (Figure 3B; eTable 4 in Supplement 1).

### Sensitivity Analyses

To assess the robustness of the present study’s findings, multiple sensitivity analyses were conducted: (1) the overlap weighting method was applied to balance baseline characteristics in the treatment and control groups instead of the inverse probability weighing approach used in the primary analyses; (2) the doubly robust approach was applied to additionally adjust for covariates in the inverse probability weighted survival models, compared with the primary approach which used inverse probability weighted survival models; (3) a high-dimensional variable selection algorithm was used to identify an additional 100 covariates that were then used, along with a predefined set of covariates, to construct the weights, compared with the primary approach that used predefined covariates; (4) the inverse probability of censoring weight was applied to account for those who died during the acute phase of infection, compared with the primary approach, in which those who died during acute phase were removed from the analyses; (5) outcomes were defined based on events that occurred 90 days after infection, compared with the primary approach, in which outcomes were defined based on events that occurred 30 days after infection; (6) incident outcome was defined as occurrence of the outcome in those without history of the related outcome within 5 years before infection, compared with the primary approach, in which the washout period was 3 years before infection; (7) hospitalization, intensive care unit admission, and ventilator use during the acute phase of infection were additionally adjusted for, compared with the primary approach that did not adjust for information after exposure; (8) outcomes were defined based on PCC *ICD-10* code (U09.9), compared with the primary definition that was based on a set of 13 predefined post–acute sequelae of COVID-19; and (9) outcomes were defined based on the first occurrence of any individual sequela, compared with the primary definition that accounted for both the number of sequelae and the influence of each sequela on health. All sensitivity analyses yielded results that are consistent (in both direction and magnitude) to those obtained using the primary approach (eTable 5 in Supplement 1).

## Discussion

In this cohort study of 281 793 people with SARS-CoV-2 infection who had at least 1 risk factor for progression to severe COVID-19 illness, compared with the control group of people who did not receive antiviral or antibody treatment during the acute phase of SARS-CoV-2 infection, treatment with nirmatrelvir within 5 days of a positive SARS-CoV-2 test was associated with reduced risk of PCC, including reduced risk of 10 of 13 post–acute sequelae examined. Nirmatrelvir was also associated with reduced risk of post–acute death and hospitalization at 180 days. Nirmatrelvir was associated with reduced risk of PCC in subgroups based on age, race, sex, smoking, cancer, cardiovascular disease, chronic kidney disease, chronic lung disease, diabetes, immune dysfunction, and hypertension. Nirmatrelvir was associated with reduced risk of PCC across strata of baseline risk, and in people who were unvaccinated, vaccinated, and boosted, and in people with primary SARS-CoV-2 infection and reinfection. Altogether, the findings suggest that treatment with nirmatrelvir during the acute phase reduces the risk of post–acute adverse health outcomes.

These results show that the salutary effect of nirmatrelvir may extend to the post–acute phase of COVID-19; nirmatrelvir was associated with reduced risk of PCC in the overall cohort and in various subgroups, including those across risk strata, vaccination status, and prior history of COVID-19. These findings are coupled with the observation that among 281 793 people with acute SARS-CoV-2 infection who had at least 1 risk factor for progression to severe disease who would be eligible for treatment with nirmatrelvir, 35 717 (12.67%) patients were treated with nirmatrelvir, and 246 076 (87.33%) patients received no antiviral treatment. The totality of evidence suggests that improving the uptake and use of nirmatrelvir in the acute phase as a means of not only preventing progression to severe acute disease but also reducing the risk of post–acute adverse health outcomes may be beneficial.

Nirmatrelvir was associated with 26% less risk of PCC, 47% less risk of post–acute death, and 24% less risk of post–acute hospitalization; the magnitude of risk reduction on the absolute scale is also substantial amounting to 4.51, 0.65, and 1.72 less cases of PCC, post–acute death, and post–acute hospitalization for every 100 treated persons between 30 to 180 days of infection. These findings should be contextualized within the broader body of evidence showing effectiveness of nirmatrelvir in also reducing risk of hospitalization or death in the acute phase.^6^ The clinical decision to initiate treatment with nirmatrelvir should consider its overall effectiveness in reducing burden of death and disease in both the acute and post–acute phases of COVID-19.

Nirmatrelvir was approved in the US for the treatment of acute COVID-19 illness in people with 1 or more risk factors for progression to severe disease. Whether the salutary benefit of nirmatrelvir extends to people without risk factors for progression to severe disease (who would not qualify for nirmatrelvir prescription under the current US Food and Drug Administration emergency use authorization and were not included in the present study’s analyses) remains to be tested in future randomized clinical trials.

We note that the present results suggested risk reduction for some but not all the prespecified post–acute sequelae in this analysis. It is possible that various sequelae are mediated by various mechanisms including some that may be affected by the receipt of antivirals and others that may not. Participants in the current study were treated in the acute phase with a 5-day course of nirmatrelvir; it remains unclear whether longer duration of treatment, a higher treatment dose, or both may have resulted in more reduced risk of post–acute sequelae. It is also unclear whether initiation of treatment in the post–acute phase of COVID-19 reduces the risk of PCC.

While we examined nirmatrelvir in this work, other antivirals that have also been shown to have efficacy and effectiveness in the acute phase (eg, molnupiravir) should also be tested to understand whether the association reported here extends to other antivirals.^27^ This approach will help expand clinicians’ armamentarium and reduce reliance on a single agent—especially with a rising risk of antiviral resistance.^28,29,30^

This study has several strengths. The VA operates the largest integrated health care system in the US, and the vast and rich national health care databases of the VA—with a large number of treated and untreated patients followed longitudinally over time—allows the evaluation of outcomes that were not assessed in randomized clinical trials. The VA data contain comprehensive information about participants, including COVID-19 testing results, medication use, vaccination records, hospitalization records, death records, and other attributes, which allows the comprehensive capture of covariates from different domains, such as demographics, diagnoses, laboratory test results, medications, vital signs, health care utilization, and contextual factors. We tested robustness of the present findings in multiple sensitivity analyses that yielded consistent results.

### Limitations

This study has several limitations. The demographic composition of the cohort (majority older, White, male adults) and accessibility to VA health care may limit generalizability of study findings. We used the electronic health care databases of the VA to conduct this study, and although we took care to adjust the analyses for a large set of predefined variables, we cannot completely rule out misclassification bias and residual confounding. We relied on filled prescription records to assign exposure, and filling of nirmatrelvir prescription does not necessarily guarantee use. We did not capture nirmatrelvir use outside the VA system; if a large number of people in the control group used nirmatrelvir outside the VA, this may bias the results toward the null. These data do not capture hospitalization and diagnoses which may have occurred outside the VA. We focused the present analyses on a prespecified set of 13 sequelae and did not examine all possible components of PCC. We examined the association of nirmatrelvir with PCC at 180 days after infection, and randomized clinical trials with longer follow-up would help further support the findings. Finally, as the virus continues to mutate, new variants emerge, and vaccine uptake improves, it is possible that the effectiveness of nirmatrelvir may also change over time.

## Conclusions

This cohort study found that in people with SARS-CoV-2 infection who had at least 1 risk factor for progression to severe disease, treatment with nirmatrelvir within 5 days of a positive SARS-CoV-2 test was associated with a reduced risk of PCC across the risk spectrum in this cohort and regardless of vaccination status and history of prior infection. These findings suggest that the salutary benefit of nirmatrelvir may extend to the post–acute phase of COVID-19.

## Notes

Supplement 1. **eFigure 1.** Cohort flow **eFigure 2.** Cohort timeline **eTable 1.** Demographic and health characteristics of the overall cohort, the nirmatrelvir group, and the control group before weighting **eTable 2.** Unadjusted event rates in nirmatrelvir and control groups **eTable 3.** Hazard ratio and absolute risk reduction of nirmatrelvir on post-acute sequelae of COVID-19, death, hospitalization, and composite outcome of death or hospitalization compared to control group **eTable 4.** Hazard ratio of nirmatrelvir on Long Covid compared to control group by subgroups **eTable 5.** Sensitivity analyses

Supplement 2.
Data sharing statement
