# Supplementary material for: Association of Treatment With Nirmatrelvir and the Risk of Post–COVID-19 Condition
Source: JAMA Intern Med. 2023 Mar 23;183(6):554–64. doi: 10.1001/jamainternmed.2023.0743 (PMC10037200; doi:10.1001/jamainternmed.2023.0743)
Supplement: Supplement 1. — eFigure 1. Cohort flow eFigure 2. Cohort timeline eTable 1. Demographic and health characteristics of the overall cohort, the nirmatrelvir group, and the control group before weighting eTable 2. Unadjusted event rates in nirmatrelvir and control groups eTable 3. Hazard ratio and absolute risk reduction of nirmatrelvir on post-acute sequelae of COVID-19, death, hospitalization, and composite outcome of death or hospitalization compared to control group eTable 4. Hazard ratio of nirmatrelvir on Long Covid compared to control group by subgroups eTable 5. Sensitivity analyses [file jamainternmed-e230743-s001.pdf]

Supplemental Online Content

Xie Y, Choi T, Al-Aly Z. Association of treatment with nirmatrelvir and the risk of post–COVID-19 condition. *JAMA Intern Med.* Published online March 23, 2023. doi:10.1001/jamainternmed.2023.0743

eFigure 1. Cohort flow

eFigure 2. Cohort timeline

eTable 1. Demographic and health characteristics of the overall cohort, the nirmatrelvir group, and the control group before weighting

eTable 2. Unadjusted event rates in nirmatrelvir and control groups

eTable 3. Hazard ratio and absolute risk reduction of nirmatrelvir on post-acute sequelae of COVID-19, death, hospitalization, and composite outcome of death or hospitalization compared to control group

eTable 4. Hazard ratio of nirmatrelvir on Long Covid compared to control group by subgroups

eTable 5. Sensitivity analyses

This supplemental material has been provided by the authors to give readers additional information about their work.

© 2023 Xie Y et al. *JAMA Internal Medicine.*

eFigure 1. Cohort flow

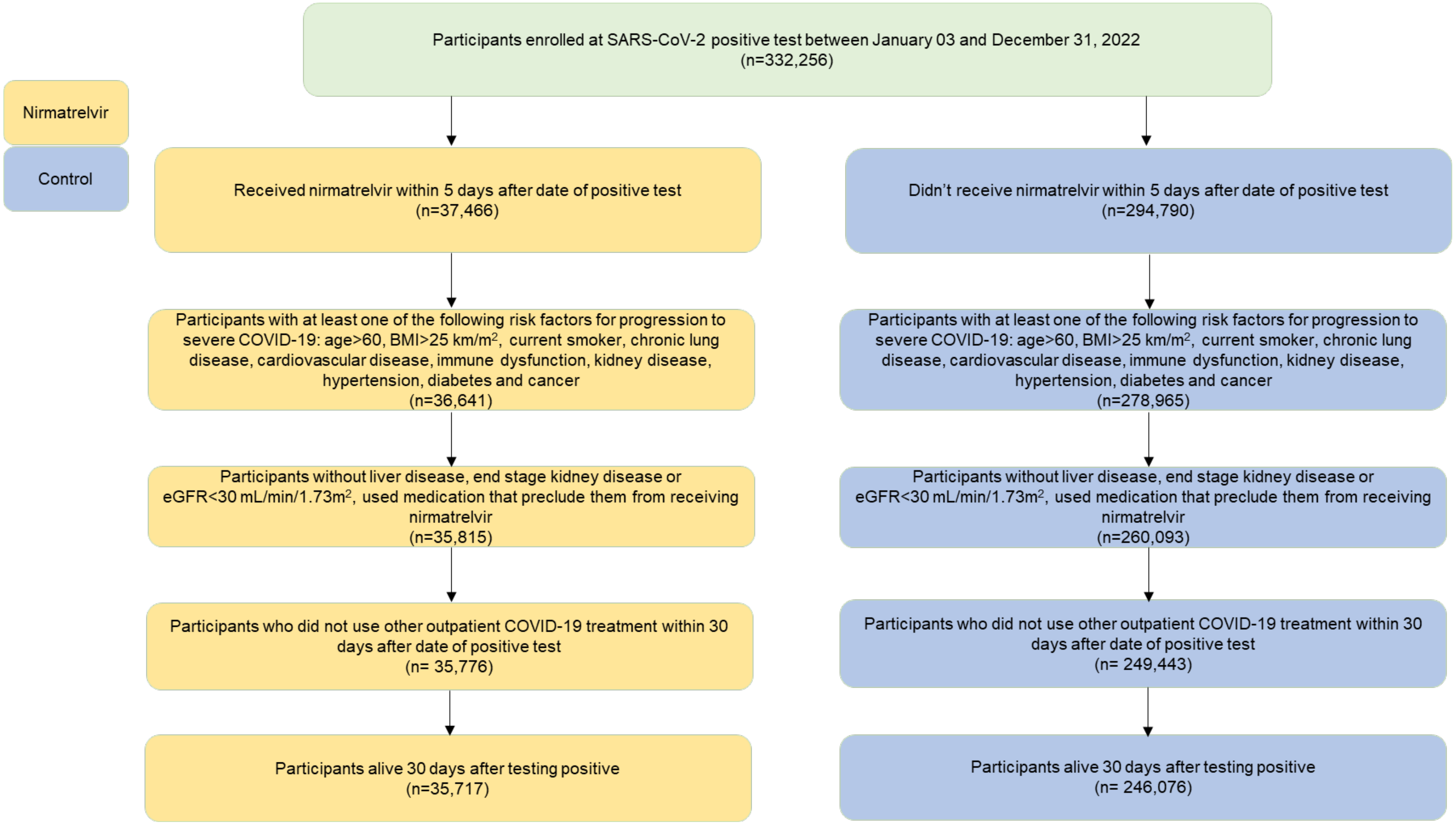

© 2023 Xie Y et al. *JAMA Internal Medicine.*

eFigure 2. Cohort timeline

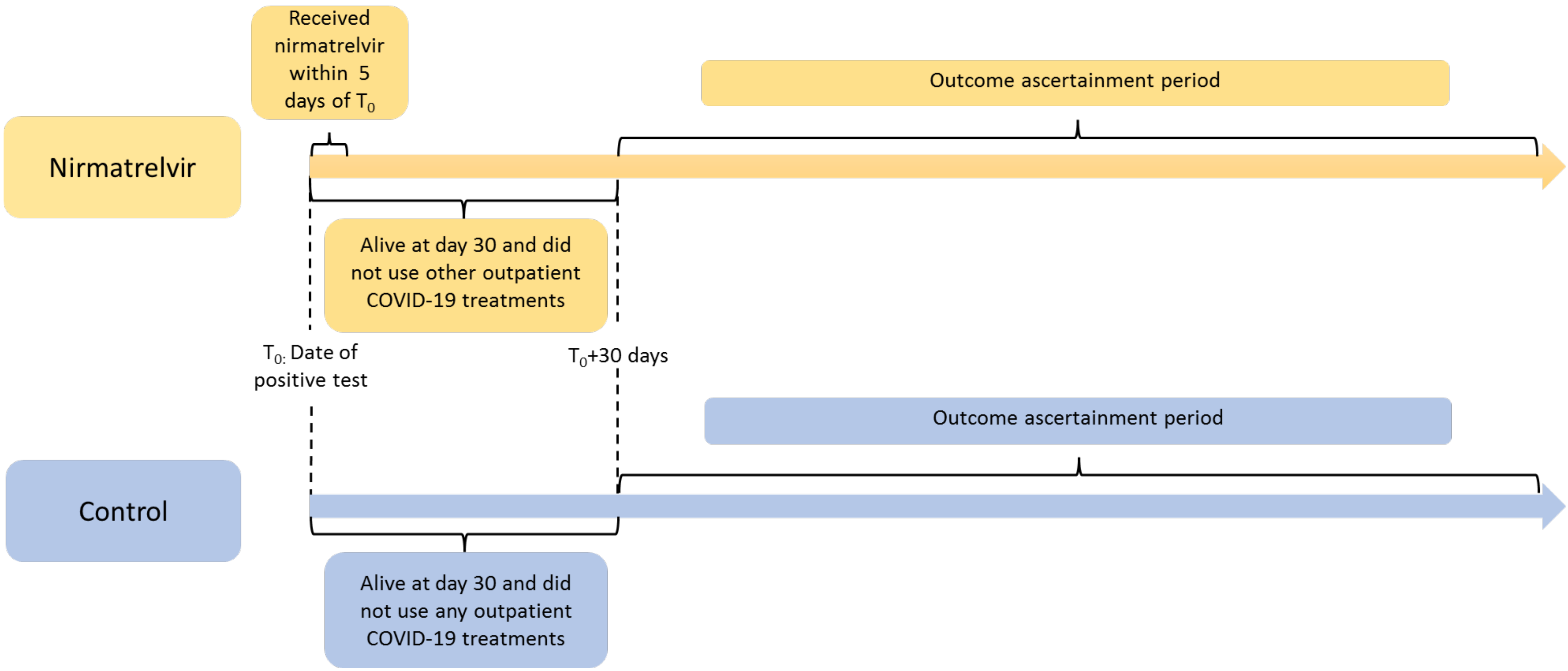

© 2023 Xie Y et al. *JAMA Internal Medicine.*

eTable 1. Demographic and health characteristics of the overall cohort, the nirmatrelvir group, and the control group before weighting

	Overall cohort N = 281,793	Control group N = 246,076	Nirmatrelvir group N = 35,717	SMD between Nirmatrelvir and control group
**Age, mean (std), yr**	61.99 (14.96)	61.46 (15.10)	65.64 (13.38)	0.29
**Race, no. (%)**				
White	201,404 (71.47)	174,973 (71.11)	26,431 (74.00)	0.07
Black	62,958 (22.34)	55,642 (22.61)	7316 (20.48)	0.05
Other	17,431 (6.19)	15,461 (6.28)	1970 (5.52)	0.03
**Ethnicity, no. (%)**				
Hispanic	25,380 (9.01)	22,557 (9.17)	2823 (7.90)	0.05
Non-Hispanic	256,413 (90.99)	223,519 (90.83)	32,894 (92.10)	0.05
**Sex, no. (%)**				
Male	242,383 (86.01)	211,044 (85.76)	31,339 (87.74)	0.06
Female	39,410 (13.99)	35,032 (14.24)	4378 (12.26)	0.06
**Smoking status, no. (%)**				
Never	128,523 (45.61)	113,053 (45.94)	15,470 (43.31)	0.05
Former	99,348 (35.26)	84,604 (34.38)	14,744 (41.28)	0.14
Current	53,922 (19.14)	48,419 (19.68)	5503 (15.41)	0.11
**Area Deprivation Index*, mean (std)**	52.74 (19.48)	52.99 (19.44)	51.07 (19.67)	0.10
**Long-term, no. (%)**	3365 (1.19)	3076 (1.25)	229 (0.64)	0.06
**Vaccination, no. (%)**				
**Without prior vaccination**	82,833 (29.39)	76,851 (31.23)	5982 (16.75)	0.34
**With 1 shots of vaccination**	15,294 (5.43)	13,998 (5.69)	1296 (3.63)	0.10
**With 2 shots of vaccination**	76,857 (27.27)	69,202 (28.12)	7655 (21.43)	0.16
**With vaccinate booster**	106,809 (37.90)	86,025 (34.96)	20,784 (58.19)	0.48
**BMI, mean (std)**	30.71 (6.19)	30.69 (6.18)	30.86 (6.31)	0.03
**eGFR, mean (std), ml/min/1.73m^2**	80.54 (19.34)	80.82 (19.46)	78.59 (18.34)	0.12
**Systolic blood pressure, mean (std), mmHg**	132.79 (11.65)	132.6 (11.69)	134.13 (11.34)	0.13
**Diastolic blood pressure, mean (std), mmHg**	78.64 (7.18)	78.68 (7.21)	78.33 (6.96)	0.05
**History of SARS-CoV-2 infection, no. (%)**	32,460 (11.52)	26,414 (10.73)	6046 (16.93)	0.18
**Use of steroid, no. (%)**	13,957 (4.95)	12,795 (5.20)	1162 (3.25)	0.10
**Medications that would have drug-drug interaction with nirmatrelvir -ritonavir**				

© 2023 Xie Y et al. *JAMA Internal Medicine.*

**On concomitant medication that require temporary hold, no. (%)**	83,553 (29.65)	65,142 (26.47)	18,411 (51.55)	0.53
**On concomitant medication that require dosing adjustment, no. (%)**	71,521 (25.38)	57,741 (23.46)	13,780 (38.58)	0.33
**On concomitant medication that require monitoring for adverse events, no. (%)**	68,072 (24.16)	54,605 (22.19)	13,467 (37.70)	0.34
**Cancer, no. (%)**	39,702 (14.09)	33,277 (13.52)	6425 (17.99)	0.12
**Chronic lung disease, no. (%)**	57,339 (20.35)	49,320 (20.04)	8019 (22.45)	0.06
**Dementia, no. (%)**	19,790 (7.02)	17,208 (6.99)	2582 (7.23)	0.01
**Diabetes mellitus type 2, no. (%)**	85,198 (30.23)	72,514 (29.47)	12,684 (35.51)	0.13
**Cardiovascular disease, no. (%)**	74,134 (26.31)	63,708 (25.89)	10426 (29.19)	0.07
**Hyperlipidemia, no. (%)**	94,745 (33.62)	80,597 (32.75)	14,148 (39.61)	0.10
**Immune dysfunction, no. (%)**	11,800 (4.19)	9790 (3.98)	2010 (5.63)	0.08
**Number of hospital admissions†, mean (std)**	0.19 (0.74)	0.19 (0.76)	0.16 (0.61)	0.04
**Number of outpatient visits†, mean (std)**	2.75 (1.55)	2.69 (1.57)	3.19 (1.39)	0.34
**Number of blood panel tests†, mean (std)**	6.94 (8.94)	6.84 (9.07)	7.63 (7.98)	0.09
**Number of medications†, mean (std)**	8.75 (7.59)	8.53 (7.61)	10.23 (7.23)	0.23
**Number of hospital admission from Medicare, mean (std)**	0.04 (0.29)	0.04 (0.30)	0.03 (0.22)	0.05
**Number of outpatient visits from Medicare, mean (std)**	0.15 (0.63)	0.15 (0.64)	0.14 (0.58)	0.02
**Influenza vaccine, no. (%)**	167,465 (59.43)	142,789 (58.03)	24,676 (69.09)	0.23
**Calendar week of study enrollment, mean (std)**	18.86 (16.11)	16.96 (15.77)	31.89 (11.85)	1.07

*. Area Deprivation Index is a measure of socioeconomic disadvantage, with a range from low to high disadvantage of 0 to 100.
†.Data collected within one year before the study enrollment.

© 2023 Xie Y et al. *JAMA Internal Medicine.*

BMI, body mass index; eGFR, estimated glomerular filtration rate; IQR, interquartile range; SMD, absolute standardized mean difference; std, standard deviation.

© 2023 Xie Y et al. *JAMA Internal Medicine.*

eTable 2. Unadjusted event rates in nirmatrelvir and control groups

Outcome	Nirmatrelvir group event rate in % at 180 days (95% CI)	Control group event rate in % at 180 days (95% CI)
**Death**	0.97 (0.86, 1.10)	2.72 (2.65, 2.79)
**Hospitalization**	7.51 (7.17, 7.86)	9.69 (9.56, 9.83)
**Death or hospitalization**	8.14 (7.79, 8.51)	11.71 (11.57, 11.86)
**Dysrhythmia**	3.63 (3.4, 3.88)	5.36 (5.27, 5.46)
**Ischemic heart disease**	1.58 (1.43, 1.75)	2.65 (2.58, 2.72)
**Pulmonary embolism**	0.53 (0.44, 0.63)	1.08 (1.03, 1.13)
**Deep vein thrombosis**	0.3 (0.24, 0.38)	0.52 (0.49, 0.55)
**Fatigue and malaise**	4.52 (4.24, 4.83)	6.45 (6.34, 6.57)
**Liver disease**	1.67 (1.51, 1.85)	2.35 (2.28, 2.42)
**Acute kidney injury**	0.94 (0.82, 1.07)	1.54 (1.49, 1.59)
**Muscle pain**	1.79 (1.63, 1.98)	2.61 (2.54, 2.68)
**Diabetes**	1.77 (1.57, 1.99)	2.58 (2.5, 2.67)
**Neurocognitive impairment**	1.61 (1.46, 1.78)	2.51 (2.45, 2.58)
**Dysautonomia**	1.25 (1.11, 1.4)	1.46 (1.41, 1.51)
**Shortness of breath**	5.35 (5.03, 5.69)	6.54 (6.42, 6.66)
**Cough**	4.62 (4.3, 4.96)	5.1 (4.99, 5.21)

Nirmatrelvir group defined as received prescription of nirmatrelvir within 5 days after tested positive for SARS-CoV-2 and did not use any other outpatient antiviral or antibody during first 30 days after tested positive.
Control group defined as did not use any outpatient antiviral or antibody during first 30 days after tested positive and served as reference group in the analyses.
Outcomes were ascertained 30 days after the SARS-CoV-2 positive test until the end of follow-up.
CI, confidence interval

© 2023 Xie Y et al. *JAMA Internal Medicine.*

eTable 3. Hazard ratio and absolute risk reduction of nirmatrelvir on Long Covid, post-acute death, hospitalization, and composite outcome of death or hospitalization compared to control group

Outcome	Relative risk (95% CI)	Nirmatrelvir group event rate in % at 180 days (95% CI)	Control group event rate in % at 180 days (95% CI)	Absolute risk reduction in % at 180 days
**Long Covid**	0.74 (0.72, 0.77)	12.99 (12.52, 13.49)	17.51 (17.08, 17.94)	4.51 (4.01, 4.99)
	**Hazard Ratio (95% CI)**	**Nirmatrelvir group event rate in % at 180 days (95% CI)**	**Control group event rate in % at 180 days (95% CI)**	**Absolute risk reduction in % at 180 days**
**Death**	0.53 (0.46, 0.61)	0.73 (0.63, 0.83)	1.38 (1.33, 1.44)	0.65 (0.54, 0.77)
**Hospitalization**	0.76 (0.73, 0.80)	5.82 (5.55, 6.09)	7.54 (7.42, 7.66)	1.72 (1.42, 2.01)
**Death or hospitalization**	0.74 (0.70, 0.77)	6.28 (6.00, 6.56)	8.43 (8.31, 8.56)	2.15 (1.85, 2.46)

Nirmatrelvir group defined as received prescription of nirmatrelvir within 5 days after tested positive for SARS-CoV-2 and did not use any other outpatient antiviral or antibody during first 30 days after tested positive.
Control group defined as did not use any outpatient antiviral or antibody during first 30 days after tested positive and served as reference group in the analyses.
Outcomes were ascertained 30 days after the SARS-CoV-2 positive test until the end of follow-up.
CI, confidence interval

© 2023 Xie Y et al. *JAMA Internal Medicine.*

eTable 4. Hazard ratio of nirmatrelvir on Long Covid compared to control group by subgroups

Subgroup	Description	Relative risk (95% CI)
Age	Age ≤ 60	0.79 (0.74, 0.85)
	60 < Age ≤ 70	0.80 (0.74, 0.87)
	Age > 70	0.66 (0.63, 0.70)
Race	White	0.72 (0.69, 0.75)
	Black	0.78 (0.72, 0.84)
Sex	Male	0.74 (0.71, 0.77)
	Female	0.71 (0.64, 0.80)
Smoking status	Current	0.71 (0.65, 0.77)
	Former	0.79 (0.74, 0.84)
	Never	0.72 (0.68, 0.76)
Cancer	No	0.74 (0.71, 0.77)
	Yes	0.77 (0.70, 0.84)
Cardiovascular disease	No	0.73 (0.69, 0.76)
	Yes	0.69 (0.65, 0.74)
Chronic kidney disease	No	0.74 (0.71, 0.77)
	Yes	0.65 (0.59, 0.71)
Chronic lung disease	No	0.74 (0.71, 0.77)
	Yes	0.75 (0.69, 0.81)
Diabetes	No	0.75 (0.72, 0.78)
	Yes	0.76 (0.71, 0.81)
Immune dysfunction	No	0.74 (0.71, 0.77)
	Yes	0.78 (0.65, 0.93)
Hypertension	No	0.74 (0.71, 0.78)
	Yes	0.74 (0.69, 0.80)
Number of baseline risk factors*	≤ 2	0.69 (0.65, 0.74)
	3 or 4	0.75 (0.71, 0.80)
	≥ 5	0.77 (0.71, 0.83)
Vaccine	Unvaccinated	0.71 (0.67, 0.76)
	1 or 2 doses of vaccine	0.74 (0.69, 0.79)
	Boosted	0.73 (0.69, 0.78)
SARS-CoV-2 infection	Primary infection	0.75 (0.72, 0.78)
	Reinfection	0.77 (0.69, 0.86)

CI, confidence interval

*. Number of baseline risk factors (1-2, 3-4 or ≥5) of progression to severe acute COVID-19 illness, where risk factors included age>60, BMI>25 km/m^2, current smoker, cancer, cardiovascular disease, kidney disease, chronic lung disease, diabetes, immune dysfunction and hypertension.

© 2023 Xie Y et al. *JAMA Internal Medicine.*

eTable 5. Sensitivity analyses

Sensitivity analyses	Relative risk (95% CI)
Balanced through overlap weighting	0.74 (0.71, 0.77)
Doubly robust adjustment	0.75 (0.72, 0.78)
Additionally adjusted for 100 high dimensional variables	0.75 (0.73, 0.78)
Applied inverse probability of censoring weight to account for those who died during the acute phase of infection	0.74 (0.72, 0.77)
Defined outcome based on events that occurred 90 days after infection	0.79 (0.76, 0.83)
Defined incident outcome in those without history of the related outcome within 5 years before infection	0.74 (0.71, 0.77)
Additionally adjusted for hospitalization, ICU admission and ventilator use during the acute phase of infection	0.88 (0.85, 0.91)
	**Hazard Ratio (95% CI)**
Defined outcome based on Long Covid ICD-10 code	0.84 (0.77, 0.92)
Defined outcome as the first occurrence of any individual sequela	0.81 (0.79, 0.84)
CI, confidence interval	

© 2023 Xie Y et al. *JAMA Internal Medicine.*
