# Supplementary material for: Association of Treatment With Nirmatrelvir and the Risk of Post–COVID-19 Condition
Source: JAMA Intern Med. 2023 Mar 23;183(6):554–64. doi: 10.1001/jamainternmed.2023.0743 (PMC10037200; doi:10.1001/jamainternmed.2023.0743)
Supplement: Supplement 2. — Data sharing statement [file jamainternmed-e230743-s002.pdf]

Data Sharing Statement

Xie. Association of Treatment With Nirmatrelvir and the Risk of Post-COVID-19 Condition. *JAMA Intern Med*. Published March 23, 2023. doi:10.1001/jamainternmed.2023.0743

Data
Data available: No

Additional Information
Explanation for why data not available: The data that support the findings of this study are available from the US Department of Veterans Affairs (VA). The VA data are made freely available to researchers behind the VA firewall with an approved VA study protocol. For more information, please visit https://www.virec.research.va.gov or contact the VA Information Resource Center (VIReC) at VIReC@va.gov. Outcome definitions provided at https://github.com/yxie618/Nirmatrelvir_PASC.
